# Neoadjuvant Talazoparib in Patients With Germline *BRCA1/2* Mutation-Positive, Early-Stage Triple-Negative Breast Cancer: Results of a Phase II Study

**DOI:** 10.1093/oncolo/oyad139

**Published:** 2023-06-15

**Authors:** Jennifer K Litton, J Thaddeus Beck, Jason M Jones, Jay Andersen, Joanne L Blum, Lida A Mina, Raymond Brig, Michael Danso, Yuan Yuan, Antonello Abbattista, Kay Noonan, Alexander Niyazov, Jayeta Chakrabarti, Akos Czibere, William F Symmans, Melinda L Telli

**Affiliations:** Department of Breast Medical Oncology, The University of Texas MD Anderson Cancer Center, Houston, TX, USA; Department of Medical Oncology and Hematology, Highlands Oncology, Springdale, AR, USA; Avera Medical Group Oncology & Hematology, Avera Cancer Institute, Sioux Falls, SD, USA; Medical Oncology, Compass Oncology, West Cancer Center, US Oncology Network, Tigard, OR, USA; Department of Oncology, Texas Oncology-Baylor Charles A. Sammons Cancer Center, US Oncology Network, Dallas, TX, USA; Hematology Oncology Department, Banner MD Anderson Cancer Center, Gilbert, AZ, USA; Medical Oncology, Brig Center for Cancer Care and Survivorship, Knoxville, TN, USA; Medical Oncology, Virginia Oncology Associates, Norfolk, VA, USA; Department of Medical Oncology & Therapeutics Research, Cedars-Sinai Cancer Center, West Hollywood, CA, USA; Clinical Statistics, Pfizer Oncology, Milan, Italy; Clinical Oncology, Pfizer Inc., Groton, CT, USA; Oncology Value & Evidence, Pfizer Inc., New York, NY, USA; Medical Oncology, Pfizer Ltd., Walton Oaks, Surrey, UK; Oncology Drug Development, Pfizer Inc., Cambridge, MA, USA; Department of Pathology, The University of Texas MD Anderson Cancer Center, Houston, TX, USA; Department of Medicine, Stanford University School of Medicine, Stanford, CA, USA

**Keywords:** poly(ADP-ribose) polymerase inhibitors, triple-negative breast neoplasms, neoadjuvant therapy, antineoplastic agents

## Abstract

**Background:**

The undetermined efficacy of the current standard-of-care neoadjuvant treatment, anthracycline/platinum-based chemotherapy, in patients with early-stage triple-negative breast cancer (TNBC) and germline *BRCA* mutations emphasizes the need for biomarker-targeted treatment, such as poly(ADP-ribose) polymerase inhibitors, in this setting. This phase II, single-arm, open-label study evaluated the efficacy and safety of neoadjuvant talazoparib in patients with germline *BRCA1/2*-mutated early-stage TNBC.

**Patients and Methods:**

Patients with germline *BRCA1/2*-mutated early-stage TNBC received talazoparib 1 mg once daily for 24 weeks (0.75 mg for moderate renal impairment) followed by surgery. The primary endpoint was pathologic complete response (pCR) by independent central review (ICR). Secondary endpoints included residual cancer burden (RCB) by ICR. Safety and tolerability of talazoparib and patient-reported outcomes were assessed.

**Results:**

Of 61 patients, 48 received ≥80% talazoparib doses, underwent surgery, and were assessed for pCR or progressed before pCR assessment and considered nonresponders. pCR rate was 45.8% (95% confidence interval [CI], 32.0%-60.6%) and 49.2% (95% CI, 36.7%-61.6%) in the evaluable and intent-to-treat (ITT) population, respectively. RCB 0/I rate was 45.8% (95% CI, 29.4%-63.2%) and 50.8% (95% CI, 35.5%-66.0%) in the evaluable and ITT population, respectively. Treatment-related adverse events (TRAE) were reported in 58 (95.1%) patients. Most common grade 3 and 4 TRAEs were anemia (39.3%) and neutropenia (9.8%). There was no clinically meaningful detriment in quality of life. No deaths occurred during the reporting period; 2 deaths due to progressive disease occurred during long-term follow-up (>400 days after first dose).

**Conclusions:**

Neoadjuvant talazoparib monotherapy was active despite pCR rates not meeting the prespecified threshold; these rates were comparable to those observed with combination anthracycline- and taxane-based chemotherapy regimens. Talazoparib was generally well tolerated.

**ClinicalTrials.gov identifier:**

NCT03499353

## Teaser

The undetermined efficacy of the current standard-of-care neoadjuvant treatment of early-stage triple-negative breast cancer (TNBC) with germline BRCA mutations emphasizes the need for biomarker-targeted treatment in this setting. This article evaluates the efficacy and safety of neoadjuvant talazoparib in patients with germline *BRCA1/2*-mutated early-stage TNBC.

**Implications for Practice**

This phase II study showed that talazoparib monotherapy was active with an observed pathologic complete response (pCR) rate of 45.8% in patients with early-stage triple-negative breast cancer and germline *BRCA1/2* mutations. Neoadjuvant talazoparib monotherapy was comparable to anthracycline- and taxane-based chemotherapy combination regimens and was generally well tolerated. Side effects were consistent with the established safety profile of talazoparib with no clinically meaningful detriment in global health status or quality of life. Multiple ongoing neoadjuvant studies may further clarify the optimal use of poly(ADP-ribose) polymerase (PARP) inhibitors in this setting.

## Introduction

Despite the overall progress in breast cancer treatments, some patients continue to have a high risk of recurrence and death after systemic therapy.^1^ Mutations in *BRCA1/2* account for approximately 30% of hereditary breast cancer and approximately 5% of all breast cancers.^2-4^ Germline mutations in *BRCA1/2* increase the probability of developing breast cancer during a lifetime to >70%.^4,5^*BRCA1/2* are tumor suppressor genes that mediate the repair of DNA double-strand breaks via homologous recombination repair (HRR).^6^ Patients with germline *BRCA1* mutations have a predisposition for triple-negative breast cancer (TNBC). In one analysis, approximately 70% of patients with *BRCA1* mutations and 16% of patients with *BRCA2* mutations had TNBC.^7^

Cancer cells with germline *BRCA1/2* mutations rely on poly(ADP-ribose) polymerase (PARP) enzymes 1 and 2 for DNA repair.^8^ PARP inhibitors inhibit PARP1/2 catalytic activity and trap PARP to single-strand breaks inducing cell death via synthetic lethality.^6^ This supports the rationale for the use of PARP inhibitors in the neoadjuvant setting for patients with germline *BRCA1/2* mutations.

Neoadjuvant chemotherapy has become a standard approach for most patients with early-stage TNBC,^9,10^ including patients with *BRCA1/2* mutations.^11^ The efficacy of neoadjuvant platinum chemotherapy for patients with germline *BRCA* mutations remains unclear,^12^ despite several studies demonstrating the efficacy of platinum-containing regimens for TNBC in the neoadjuvant setting,^13,14^ including the KEYNOTE-522 study, which evaluated paclitaxel-, carboplatin-, and anthracycline-based chemotherapy with either pembrolizumab or placebo.^10^ Lack of clear benefit has been demonstrated in both the INFORM study, in which 70% of patients had TNBC, and the GeparSixto study, in which 54% of patients had TNBC and showed that patients with germline *BRCA* mutations did not receive further benefit from the addition of platinum.^15,16^ These results highlight the need to investigate targeted therapies for patients with TNBC and germline *BRCA* mutations.

In the phase III BrighTNess study, the PARP inhibitor veliparib plus carboplatin in addition to weekly neoadjuvant paclitaxel followed by doxorubicin and cyclophosphamide in patients with TNBC demonstrated a clinically significant improvement of pathologic complete response (pCR) vs. paclitaxel alone followed by doxorubicin and cyclophosphamide (*P* < .0001).^9^ However, the addition of veliparib to carboplatin/paclitaxel combination did not appear to add significant benefit compared with carboplatin/paclitaxel alone.^9^ This study failed to demonstrate an improvement in pCR from adding PARP inhibition to standard of care therapy but did not evaluate the use of single-agent PARP inhibition as a neoadjuvant strategy by itself.^9^

Talazoparib is a PARP inhibitor approved in the United States, the European Union, and multiple other countries as monotherapy for the treatment of patients with deleterious or suspected deleterious germline *BRCA-*mutated, human epidermal growth factor receptor 2 (HER2)-negative, locally advanced or metastatic breast cancer.^17,18^ Talazoparib is the first single-agent targeted therapy to achieve pCR in germline *BRCA*-positive, HER2-negative patients with early breast cancer, including TNBC.^19^ Results from a prior investigator-initiated study (NCT02282345) showed that of 19 patients enrolled with early-stage I to III breast cancer and germline *BRCA* mutations with pathologic response data, 53% (95% confidence interval [CI], 32%-73%) achieved pCR and 63% (95% CI, 41%-81%) achieved residual cancer burden (RCB) 0/I with neoadjuvant talazoparib monotherapy for 24 weeks followed by surgery.^19^ The most common adverse events (AEs) observed in this treatment-naive patient population were anemia and nausea, which were consistent with those previously reported with PARP inhibitors in the metastatic breast cancer setting.^19-21^ Moreover, these early results provided the rationale for the current confirmatory phase II study in a larger patient population (NEOTALA; NCT03499353).

## Methods

### Study Design and Patients

**Figure 1.**
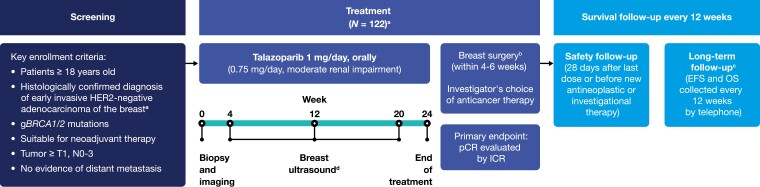
Study design. Abbreviations: BC, breast cancer; EFS, event-free survival; g*BRCA1/2*, germline breast cancer susceptibility genes 1 and 2; HER2, human epidermal growth factor receptor 2; HR, hormone receptor; ICR, independent central review; N, regional lymph nodes; OS, overall survival; pCR, pathologic complete response; T, primary tumor. ^a^Study design was amended to include HR-positive, HER2-negative patients with BC and the patient numbers were reduced from 122 to 60 to address lower than expected enrollment. ^b^Breast/axillary tumor tissue must be removed by either lumpectomy or mastectomy with clinically appropriate axillary surgery. Patients who had disease progression were to discontinue treatment on study and switch to alternative systemic therapy or go straight to surgery. ^c^Long-term follow-up was planned to be at least 3 years, starting from the date of surgery for EFS and after the first dose of drug for OS. However, Pfizer decided to make a strategic change in the development program for talazoparib in neoadjuvant BC and decided not to pursue further development in this setting. The study was closed after all patients completed safety follow-up, and EFS/OS was not reached. ^d^Breast ultrasound scans at week 12 were used to assist in clinical response assessment during treatment and to provide investigator reassurance that the patient was not progressing. Breast ultrasound was not used to assess response.

NEOTALA was an open-label, multicenter, single-arm, phase II study initially designed to enroll 122 patients (Fig. 1). However, due to lower-than-expected recruitment rate, target sample size was reduced to 60 adult patients with a histologically confirmed diagnosis of early breast cancer with hormone receptor (HR)-negative, HER2-negative disease and germline *BRCA1/2* mutations (Fig. 1). Although inclusion criteria changed during the course of the study to allow HR-positive patients (Amendment 4; August 14, 2019), no HR-positive patients were enrolled by the time the study closed to new patients in March 2020. Further key inclusion criteria included patients ≥18 years of age, adenocarcinoma of the breast, germline *BRCA1/2* mutations, tumors ≥T1, N0-3, no evidence of distant metastasis, and those suitable for neoadjuvant therapy (Fig. 1). All patients had *BRCA* mutation status determined via screening (BRACAnalysis CDx^®^ [Myriad Genetics, Salt Lake City, Utah]) or historical evidence of a *BRCA1/2* mutation by Myriad BRACAnalysis CDx^®^ post 2016. Key exclusion criteria included any previous antitumor therapies for the current breast cancer event, previous or concomitant systemic anticancer therapies used for the treatment of cancer in the last 3 years, prior treatment with a PARP inhibitor in any disease setting, and patients with myelodysplastic syndrome (MDS).

In keeping with the initial pilot study, the prescribed treatment phase was 24 weeks (6 cycles of 4 weeks). Patients were administered a starting dose of oral talazoparib 1 mg/day, or 0.75 mg/day for moderate renal impairment. Treatment was followed by breast surgery within 6 weeks of completing neoadjuvant treatment, and pCR was assessed by both independent central review (ICR) and investigator. Standard-of-care treatment could be initiated following progressive disease (PD) with investigator’s choice of therapy. Investigator’s choice of systemic therapy could also be initiated post-surgery. The safety follow-up period was 28 days after the last dose of talazoparib or initiation of a new anticancer therapy. Long-term follow-up of event-free survival (EFS), defined as the time from surgery date to first documentation of local or distant recurrence, death, or initiation of antineoplastic therapy before documentation of first relapse, assessed starting after surgery, and overall survival (OS), assessed from first dose, was planned for 3 years, and collected every 12 weeks by telephone.

Because of a strategic change in the development program by the sponsor, not related to safety or efficacy concerns for talazoparib in the neoadjuvant breast cancer setting, the study was closed on September 23, 2020, after all patients completed the safety follow-up, and therefore, the long-term EFS and OS endpoints were not met or evaluated.

The study protocol was reviewed and approved by an institutional review board and ethics committee and research ethics board, and conducted under the International Ethical Guidelines for Biomedical Research Involving Human Patients, the International Council for Harmonisation Guideline for Good Clinical Practice, the Declaration of Helsinki, and other applicable national and local regulations. Written evidence of informed consent was required before patients could be enrolled into the study.

### Endpoints

The primary endpoint was pCR in the evaluable population assessed by ICR, and defined as the absence of invasive cancer in the breast and axillary lymph nodes on H&E staining of the complete resected breast specimen, and all sampled regional lymph nodes following completion of neoadjuvant systemic therapy (ie, ypT0/Tis ypN0 in the current American Joint Committee on Cancer staging system).^22^ The evaluable population was the primary analysis population and consisted of patients who received ≥80% of the planned talazoparib doses prescribed at treatment initiation, underwent breast/axillary surgery, and had a pCR assessment. A reduction in relative dose intensity (RDI) below 80% is considered a clinically significant reduction from planned therapy and maintaining RDI has been associated with improved survival, hence in this study a cut-off of 80% was chosen.^23^ In addition, patients who progressed before pCR could be assessed were included in the evaluable population (and considered as nonresponders).

Isolated tumor cells (ITCs) were not specifically collected, therefore, no data on ITCs are available. Key secondary endpoints included pCR rate in the intent-to-treat (ITT) population assessed by ICR, and pCR by investigator assessment in both ITT and evaluable populations as well as RCB by ICR in both populations. All patients who took ≥1 dose of talazoparib were included in the ITT population and evaluated for efficacy and safety.

RCB is a 4-category index derived from primary tumor dimensions, cellularity of the tumor bed, and axillary nodal burden. RCB 0 refers to no residual invasive cancer, or pCR. RCB I refers to minimal RCB, RCB II to moderate RCB, and RCB III to extensive RCB (PD).^24^ Only patients who received surgery and histologically assessed by ICR were evaluated for RCB. Safety and tolerability of talazoparib were also assessed. EFS and OS were to be assessed at 3 years.

### Patient-Reported Outcome Endpoints

Patient-reported outcomes (PROs) were measured at baseline, and every 4 weeks for 24 weeks or at disease progression via the European Organisation for Research and Treatment of Cancer (EORTC) Quality of Life Questionnaire (QLQ-C30), the EORTC breast cancer module (QLQ-BR23), and the European Quality of Life 5-Domain 5-Level Scale (EQ-5D-5L) health questionnaire. Missed expected menstrual periods were assessed via the Patient-Reported Outcomes version of the Common Terminology Criteria for Adverse Events (PRO-CTCAE) questionnaire.

The EORTC QLQ-C30 is a standardized instrument that assesses cancer-specific patient-reported global quality of life, functioning, and disease/treatment-related symptoms. Patients self-rate their self-care, activity level, pain/discomfort, and mental health during the past week by choosing responses from a 4-point Likert scale. On all EORTC scales, responses to all items are converted to a 0-to-100 scale with a standard scoring algorithm. For functional and Global Health Status/Quality of Life (GHS/QoL) scales, higher scores indicate a better level of functioning and quality of life. For symptom scales, a higher score indicates greater symptom severity.

The EQ-5D-5L consists of 5 dimensions (mobility, self-care, usual activities, pain/discomfort, and anxiety/depression), each of which have 5 possible responses indicating the level of the problem (1, no problem; 2, slight problem; 3, moderate problem; 4, severe problem; 5, extreme problem). Results of the 5 dimensions are combined to derive an EQ-5D index score that ranges from −0.594 to 1.0 corresponding to worst and best health status (using a UK valuation based on the EQ-5D-3L).^25^

The PRO-CTCAE item library questionnaire is a PRO measure developed to evaluate symptomatic toxicity in patients on cancer clinical trials.

### Statistical Analysis

The study was to be considered a success if the posterior probability that the true pCR rate exceeds 45% was ≥0.8. An interim analysis was to be conducted once 28 evaluable patients were assessed for pCR, and if ≥12 responses were observed, the study was to continue enrolling up to 60 patients.

The pCR rate was defined as the percentage of patients achieving pCR after talazoparib treatment in both the evaluable and ITT populations by ICR and investigator, and related exact 2-sided 80% and 95% CIs were based on the Blaker’s method. RCB was calculated by ICR in both the evaluable and ITT populations, along with the corresponding exact 2-sided 95% CIs based on the Goodman’s method. Safety and tolerability of talazoparib were evaluated in the ITT population.

Prespecified PRO analyses included the overall mean change from baseline (estimated using the longitudinal mixed-effects model) and the time to clinically meaningful deterioration (summarized using Kaplan-Meier methods). The time to clinically meaningful deterioration according to the GHS/QoL scale was defined as the first observation with a decrease of ≥10 points and no subsequent observations with a decrease of <10 points from baseline based on previously established thresholds.^26^ Similarly, time to deterioration according to the nausea/vomiting symptoms scale was defined as the first observation with an increase of ≥10 points and no subsequent observations with an increase of <10 points from baseline. Missed expected menstrual periods are reported descriptively as the proportion of patients having ≥1 missing menstrual period over the 6 cycles.

## Results

### Patient Disposition

**Figure 2.**
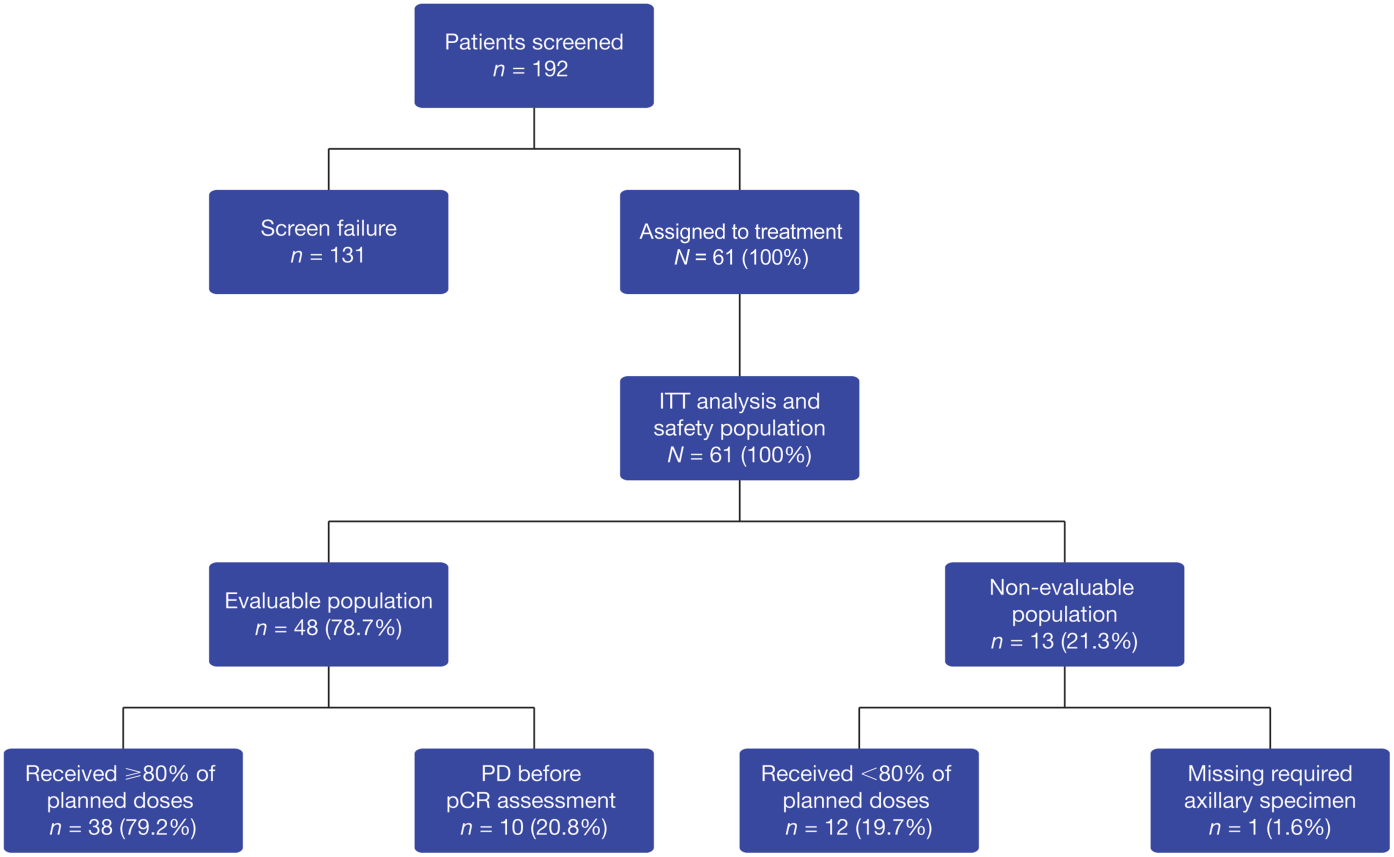
Patient populations. Abbreviations: ITT, intent-to-treat; pCR, pathologic complete response; PD, progressive disease.

Between August 27, 2018, and February 5, 2020, a total of 192 patients in the United States were screened (including for germline *BRCA1/2* mutations) and 61 patients were treated (ITT population); 48 patients were included in the evaluable population (Fig. 2), and 13 patients were considered non-evaluable, 12 of whom received <80% of planned doses, and 1 patient did not have surgery at the investigator site. Of the 12 patients who received <80% of planned doses, 10 had AEs leading to dose interruption or dose reduction, one was not compliant with treatment administration and decided to discontinue treatment, and one withdrew from the study. The study results met the threshold for the continuation of enrollment (13 pCR out of the first 28 evaluable patients). Forty-five patients completed the neoadjuvant treatment phase with talazoparib, and 49 patients completed the surgical safety follow-up phase (Supplementary Table S1). Fifty-eight patients entered the long-term follow-up phase, of whom 55 (90.2%) patients discontinued due to study closure, 2 patients died due to PD (>400 days after first dose), and 1 patient withdrew consent (Supplementary Table S1).

### Baseline Patient Characteristics

All patients were female with a median age of 42 years and an average duration since onset of breast cancer of 4.5 weeks (Table 1). Thirty-six (59%) patients were premenopausal and 25 (41%) patients were postmenopausal, with the majority of patients being White (77.0%), Black/African American (11.5%), or Asian (4.9%). All women were diagnosed with TNBC with 78.7% germline *BRCA1*-positive and 21.3% germline *BRCA2*-positive (Table 1). Of the 61 patients in the ITT population, 60 patients had adenocarcinoma and 1 patient had squamous carcinoma with spindle cell features.

**Table 1.** Baseline patient characteristics (ITT population).

Patient baseline characteristic	Talazoparib (*N* = 61)
Age (years)	
Median (min-max)	42 (26-75)
Sex, *n* (%)	
Female	61 (100.0)
Menopausal status, *n* (%)	
Premenopausal	36 (59.0)
Postmenopausal	25 (41.0)
Race, *n* (%)	
White	47 (77.0)
Black or African American	7 (11.5)
Asian	3 (4.9)
Not reported	4 (6.6)
Ethnicity, *n* (%)	
African American	7 (11.5)
Ashkenazi Jewish	1 (1.6)
Chinese	1 (1.6)
Other^a^	52 (85.2)
Hispanic or Latino	3 (4.9)
Not Hispanic or Latino	42 (68.9)
Not reported	7 (11.5)
Breast cancer	
*Duration since onset (weeks)*	
Mean (min-max)	4.54 (0.4-21.1)
Adenocarcinoma, *n* (%)	60 (98.4)
Squamous carcinoma with spindle cell, *n* (%)	1 (1.6)
TNBC, *n* (%)	61 (100.0)
* BRCA1*, *n* (%)	48 (78.7)
* BRCA2*, *n* (%)	13 (21.3)
Staging, *n* (%)	
Stage I	20 (32.8)
Stage II	27 (44.3)
Stage III	14 (23.0)

Abbreviations: *BRCA1/2*, breast cancer susceptibility genes 1 and 2; ITT, intent-to-treat; max, maximum; min, minimum; TNBC, triple-negative breast cancer.

^a^“Other” includes 3 patients who were not recorded in the “Other” category (designation left blank) but reported their ethnicity as Hispanic or Latino (*n* = 1), Not Hispanic or Latino (*n* = 1) and Not Reported (*n* = 1)

### Pathologic Complete Response

**Figure 3.**
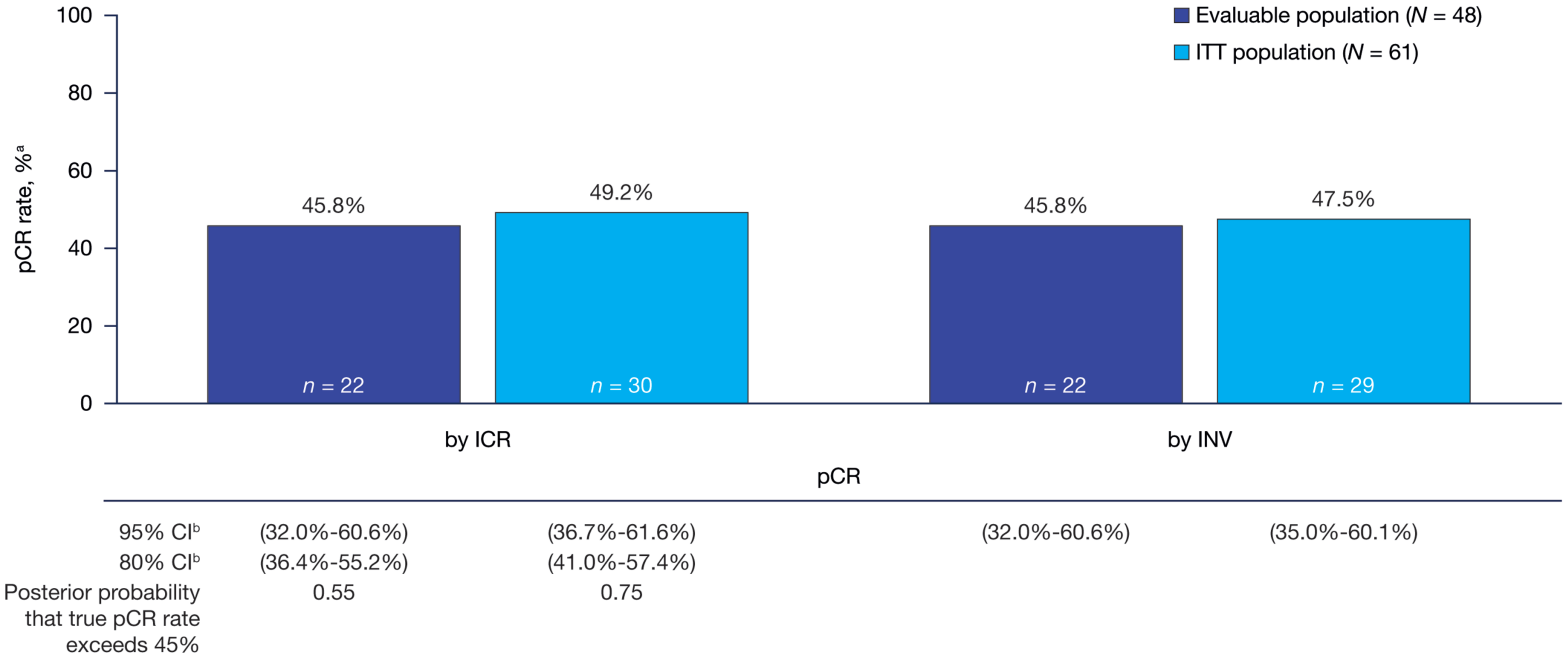
Pathologic complete response. Abbreviations: CI, confidence interval; ICR, independent central review; INV, investigator; ITT, intent-to-treat; pCR, pathologic complete response. ^a^The denominator is *N*, the number of patients in the evaluable/ITT analysis set as per ICR/INV. ^b^The exact CI was calculated using the Blaker’s method.

Twenty-two (45.8%) of 48 patients in the evaluable population achieved pCR (95% CI, 32.0%-60.6%) by both ICR and investigator assessment. Among patients in the ITT population, the pCR rate was 49.2% (30/61) (95% CI, 36.7%-61.6%) and 47.5% (29/61) (95% CI, 35.0%-60.1%) by ICR and investigator assessments, respectively (Fig. 3). Nine patients in the ITT population who achieved pCR had breast cancer stage I at initial diagnosis, 11 patients had stage II, and 10 patients had stage III/other (Supplementary Table S2). Of the 12 patients who received <80% of the planned treatment, 8 achieved pCR. The posterior probability that true pCR rate exceeds 45% was 0.55 in the evaluable population and 0.75 in the ITT ­population. The prespecified threshold was not passed (corresponding to 25 pCR in 48 evaluable patients and 31 in 61 ITT patients).

### Residual Cancer Burden

**Figure 4.**
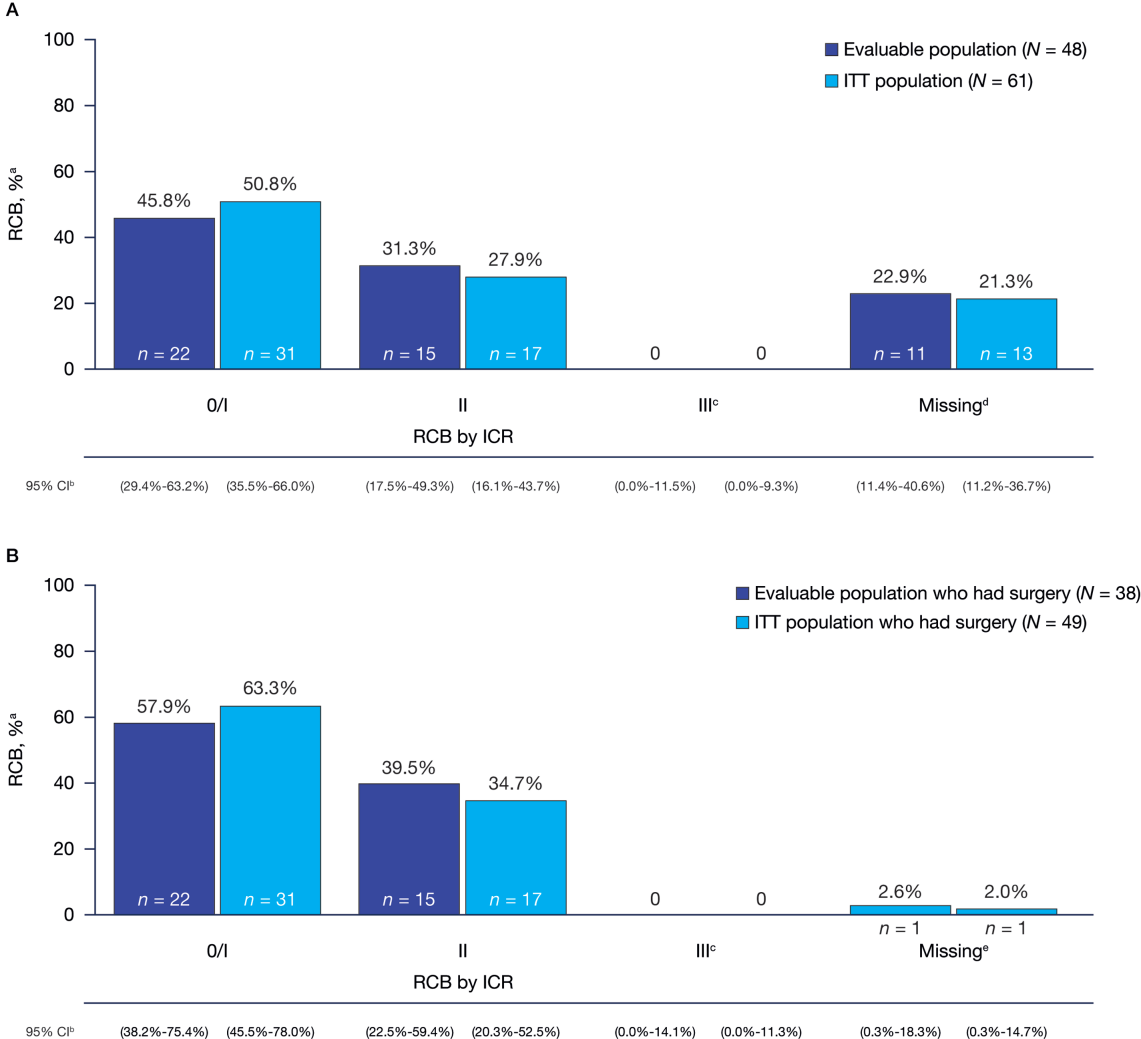
**(A)** Residual cancer burden (all patients); **(B)** Residual cancer burden among patients who had surgery. Abbreviations: CI, confidence interval; ICR, independent central review; ITT, intent-to-treat; PD, progressive disease; RCB, residual cancer burden. ^a^The denominator is *N*, the number of patients in the evaluable/ITT analysis set as per ICR. ^b^The simultaneous exact CI was calculated using Goodman’s method. ^c^None of the patients who had pathology submitted are classified as RCB III. ^d^10 patients in the evaluable and ITT population had PD and are counted in the “Missing” category. Two patients did not have surgery for other reasons (early discontinuation and consent withdrawal; ITT population), and 1 patient was unable to be assessed because of missing a required axillary specimen. ^e^One patient was unable to be assessed because of missing a required axillary specimen.

Among 48 patients in the evaluable population, 45.8% (*n* = 22; 95% CI, 29.4%-63.2%) achieved RCB 0/I by ICR compared with 50.8% (*n* = 31/61; 95% CI, 35.5%-66.0%) in the ITT population (Fig. 4A). Fifteen patients (31.3%; 95% CI, 17.5%-49.3%) in the evaluable population and 17 patients (27.9%; 95% CI, 16.1%-43.7%) in the ITT population had RCB II. A summary of breast cancer stage at initial diagnosis by RCB in the ITT population is presented in Supplementary Table S2. Ten patients had PD among the evaluable and ITT population, of whom 2 patients had stage I, 6 patients had stage II, and 2 patients had stage III breast cancer at initial diagnosis. Patients who had progression during study treatment and switched to chemotherapy were not assessed by ICR for RCB and are counted in the “Missing” category. However, per Symmans et al. (2017), these 10 patients would have been considered RCB III (Fig. 4A).^24^

Among patients who had surgery (*n* = 38 evaluable ­population; *n* = 49 ITT population), 57.9% (*n* = 22; 95% CI, 38.2%-75.4%) of the evaluable population compared with 63.3% (*n* = 31; 95% CI, 45.5%-78.0%) of the ITT population achieved RCB 0/I (Fig. 4B). RCB II was found in 15 (39.5%; 95% CI, 22.5%-59.4%) patients in the evaluable population and in 17 (34.7%; 95% CI, 20.3%-52.5%) patients in the ITT population.

### Drug Exposure

Mean talazoparib exposure was 23.3 weeks, and 90.2% (*n* = 55) of patients received talazoparib for ≥20 weeks (Supplementary Table S3). Patients with dose interruptions due to toxicities could make up missed doses per investigator discretion extending the patient’s treatment longer than 24 weeks. Forty-five (73.8%) patients received talazoparib for ≥24 weeks. The mean overall RDI was 84.5% (Supplementary Table S3).

### Safety

In total, 58 (95.1%) patients experienced treatment-related AEs (TRAEs) (Table 2). The most common TRAEs ≥10% were fatigue (*n* = 47; 77%), nausea (*n* = 39; 63.9%), and alopecia (*n* = 35; 57.4%). Most TRAEs were grade 1 or 2. Most common grade 3 and 4 TRAEs were anemia (*n* = 24; 39.3%) and neutropenia (*n* = 6; 9.8%). No cases of MDS were reported.

**Table 2.** The most common treatment-related adverse events experienced by ≥10% of patients (ITT population).

Number (%) of patients by preferred term^a^	Talazoparib (*N* = 61)				
	Grade 1	Grade 2	Grade 3	Grade 4	Total
Any adverse event	22 (36.1)	9 (14.8)	26 (42.6)	1 (1.6)	58 (95.1)
Fatigue	34 (55.7)	12 (19.7)	1 (1.6)	0	47 (77.0)
Nausea	31 (50.8)	7 (11.5)	1 (1.6)	0	39 (63.9)
Alopecia	33 (54.1)	2 (3.3)	0	0	35 (57.4)
Anemia	4 (6.6)	1 (1.6)	24 (39.3)	0	29 (47.5)
Headache	16 (26.2)	2 (3.3)	1 (1.6)	0	19 (31.1)
Dizziness	11 (18.0)	1 (1.6)	0	0	12 (19.7)
Constipation	9 (14.8)	2 (3.3)	0	0	11 (18.0)
Neutrophil count decreased	1 (1.6)	2 (3.3)	5 (8.2)	1 (1.6)	9 (14.8)
White blood cell count decreased	5 (8.2)	3 (4.9)	1 (1.6)	0	9 (14.8)
Decreased appetite	7 (11.5)	1 (1.6)	0	0	8 (13.1)
Diarrhea	6 (9.8)	2 (3.3)	0	0	8 (13.1)

Abbreviations: CTCAE, Common Terminology Criteria for Adverse Events; ITT, intent-to-treat; MedDRA, Medical Dictionary for Regulatory Activities; NCI-CTCAE, National Cancer Institute Common Terminology Criteria for Adverse Events.

^a^Includes all data collected since the first dose of study drug. If the same patient had more than 1 occurrence in the same preferred term event category, only the occurrence with maximum CTCAE grade is counted. Patients are counted only once per event. MedDRA v23.1 coding dictionary applied. NCI-CTCAE version 4.03.

### Patient-Reported Outcomes

#### EORTC QLQ-C30

Based on the repeated measures mixed-effect model, there was no clinically meaningful overall change from baseline in GHS/QoL (estimated mean −9.44; 95% CI, −13.11 to −5.77). The median time to definitive deterioration (TTD) was not estimable, and the 6-month probability of not experiencing a definitive deterioration in GHS/QoL was 59.4%. Based on the repeated measures mixed-effect model, there was no clinically meaningful overall change from baseline in nausea/vomiting (estimated mean 7.36; 95% CI, 4.67-10.05). The median TTD was not estimable, and the 6-month probability of not experiencing a definitive deterioration in nausea/vomiting was 81.8%.

#### EQ-5D-5L

Based on the repeated measures mixed-effect model, there was no clinically meaningful change from baseline^27^ in EQ-5D index score (estimated mean −0.02; 95% CI, −0.04 to 0.00).

### PRO-CTCAE Missed Menstrual Period

Of the 32 premenopausal patients, 6 (18.8%) patients reported missing at least 1 menstrual period in the last 7 days across the 6 cycles.

### Dose Modifications and Supportive Care

In the event of a grade 3 or greater toxicity, daily dosing was interrupted. Upon resuming treatment, talazoparib was administered at the next lower dose level (reduced increments of 0.25 mg/day). Twenty (32.8%) patients had AEs leading to dose interruptions and 24 (39.3%) had AEs leading to dose reductions (Table 3).

**Table 3.** Dose modifications and permanent drug discontinuation (ITT population).

Dose interruptions, reductions, transfusions, and discontinuations	Talazoparib (*N* = 61)
Number (%) of patients	***n* (%)**
Dose interruptions due to AEs	20 (32.8)
Dose reductions due to AEs	24 (39.3)
Packed red blood cell transfusion	13 (21.3)
Transfusion	7 (11.5)
Permanent drug discontinuation	
Adverse event	3 (4.9)
Death	0
Progressive disease	10 (16.4)
Withdrawal by patient^a^	2 (3.3)
Other^b^	1 (1.6)

Abbreviations: AE, adverse event; ITT, intent-to-treat.

^a^One patient completed only 4 months of treatment and decided to have surgery early and 1 patient withdrew consent from the treatment and also permanently discontinued from the study.

^b^Patient had surgery early in another country and specimen was not provided to central laboratory.

Anemia is a known AE for talazoparib. In this study, 24 patients (39.3%) experienced treatment-related grade 3 anemia (Table 2). Median time from the first dose of talazoparib to the onset of the first grade 3 anemia event was 85 days (range: 41-141). The earliest onset of grade 3 anemia was at the end of cycle 2; however, most grade 3 anemia events started in either cycle 3 (*n* = 9) or cycle 4 (*n* = 11). Nine patients had recurrent grade 3 anemia during the treatment period. Twenty (32.8%) patients received packed red blood cell (PRBC)/blood transfusions due to grade 3 anemia. Eight (13.1%) patients received 1 unit of PRBCs; 2 (3.3%) patients received 2 units; 3 (4.9%) patients received 3 units; 4 (6.6%) patients received 4 units; 1 (1.6%) patient received 5 units; and 2 (3.3%) patients received 8 units. Of the patients with grade 3 anemia (*n* = 24), the majority received PRBC/blood transfusions within one day of onset and all patients had initial dosing interruptions/reductions as defined by the protocol dose-modification guidance. Four patients had more than 1 dose interruption, 7 patients had dose reductions, and 2 patients discontinued talazoparib because of recurrent grade 3 anemia.

### Discontinuation and Long-term Follow-up

Sixteen (26.2%) patients discontinued study treatment before completion of the 24-week period: 3 patients discontinued due to AEs, 10 patients discontinued due to PD, 2 patients withdrew consent, and 1 patient discontinued early to have surgery (Table 3).

In the long-term follow-up phase, 4/30 (13.3%) patients with pCR, 10/16 (62.5%) patients with pathologic partial response (pPR), and 2/2 (100%) patients with no response in the ITT population received a combination of adjuvant cyclophosphamide, doxorubicin, platinum, or taxane-based therapy. In addition, of the 10/16 patients with pPR who received adjuvant therapy, 2 patients received capecitabine and 1 patient each received fluorouracil and zoledronic acid.

Of the 10 patients who experienced PD, 5 patients received additional neoadjuvant chemotherapy within 1-8 days from the date of PD. Of the 5 patients who received neoadjuvant chemotherapy, all received platinum therapy, 4 patients received paclitaxel, and 4 patients received cyclophosphamide and doxorubicin. Among the 4 patients with PD who received adjuvant chemotherapy following surgery, patients received taxane, cyclophosphamide, pertuzumab, ­trastuzumab, atezolizumab, antimetabolite, and/or platinum therapy. One patient received both additional neoadjuvant and adjuvant chemotherapy.

## Discussion

The results from the NEOTALA study showed that talazoparib monotherapy was active, despite not meeting the predefined threshold, and was well tolerated in the neoadjuvant setting. The pCR rate by ICR was 45.8% (95% CI, 32.0%-60.6%) and 49.2% (95% CI, 36.7%-61.6%) in the evaluable and ITT analysis population, respectively, and comparable with pCR rates previously observed with neoadjuvant ­chemotherapy regimens with combination anthracycline- and taxane-based chemotherapy regimens.^9^ Furthermore, the pCR rates were comparable with a phase I pilot study of neoadjuvant niraparib monotherapy where 40% (*n* = 6/15) of patients with *BRCA* mutations and TNBC achieved pCR.^28^ While efficacy is typically higher in the evaluable population compared to the ITT population, the pCR rate in the NEOTALA study was greater in the ITT population since 8 patients were considered non-evaluable as they received <80% of the planned talazoparib dose but still achieved pCR. Thus, despite the fact a decrease in RDI below 80% is considered a clinically significant reduction from standard therapy,^23^ the results of the NEOTALA study potentially suggest a shorter neoadjuvant treatment duration in future studies, although further investigation in larger patient populations is warranted.

In this study, 10 (16.4%) patients experienced PD with ­talazoparib, which may reflect PARP inhibitor resistance in these patients. In the phase II PETREMAC study, which evaluated neoadjuvant olaparib monotherapy, no patients with primary TNBC and *BRCA* mutations experienced PD.^29^ However, the lower PD rate achieved with neoadjuvant olaparib may be explained by the small patient population with g*BRCA1/2* mutations (*n* = 4) enrolled in the study. PARP inhibitor resistance can occur through various mechanisms, including reversion of mutated *BRCA* genes,^30^ HRR restoration, and DNA replication fork protection, which reduce PARP inhibitor sensitivity and thus negatively impact response.^31,32^ Nonetheless, further research is required to understand how these mechanisms translate to PARP inhibitor resistance observed in the clinic.^31-33^

TRAEs were consistent with the established safety profile of talazoparib^20,21^ with no clinically meaningful detriment in GHS/QoL or nausea/vomiting. Twenty (32.8%) patients had dose interruptions for AEs. Most common TRAEs included fatigue, nausea, alopecia, and anemia. Twenty-four patients (39.3%) experienced treatment-related grade 3 anemia with a median time to onset of 85 days. Twenty (32.8%) patients received transfusions to support anemia, which is comparable with previous talazoparib safety studies in the advanced/metastatic setting including the ABRAZO and EMBRACA studies in which PRBC transfusions were administered to 28% and 38% of patients, respectively.^20,21^

NEOTALA was limited by its non-randomized, single-arm design, small sample size, and early termination due to sponsor decision. In addition, response assessment per breast ultrasound scan was non-standardized so could not be determined.

Other clinical studies have demonstrated the utility of PARP inhibitors in this patient population. The phase III OlympiA study assessed olaparib in patients with high-risk HER2-negative germline *BRCA1/2*-mutated breast cancer randomized to 1-year adjuvant olaparib vs. placebo.^34^ The 3-year invasive, disease-free survival was greater in patients treated with olaparib vs. patients administered placebo (85.9% vs. 77.1%; hazard ratio for invasive disease or death, 0.58; 99.5% CI, 0.41-0.82; *P* < .001).^34^ Results from the OlympiA study suggest that PARP inhibitors have a substantial role in the treatment of patients with a germline *BRCA* mutation and early breast cancer. Similarly, in the GeparOLA study, paclitaxel was combined with either olaparib or carboplatin for the initial segment of neoadjuvant therapy in patients with HER2-negative breast cancer followed by epirubicin and cyclophosphamide.^35^ The primary endpoint of pCR rate for patients who received paclitaxel-olaparib was 55.1% (90% CI, 44.5%-65.3%) compared to 48.6% (90% CI, 34.4%-63.2%) for patients who received paclitaxel-carboplatin, and paclitaxel-olaparib was better tolerated.^35^

Multiple ongoing neoadjuvant studies may further clarify the optimal use of PARP inhibitors in germline *BRCA*-mutated early breast cancers. Notably, the PARTNER study (NCT03150576) is a randomized phase II/III study evaluating the addition of olaparib to platinum-based neoadjuvant chemotherapy in patients with TNBC and/or germline *BRCA*-mutated breast cancer (*N* = 756) and pCR as the primary endpoint.^36^ In addition, following FDA approval of pembrolizumab plus platinum-containing neoadjuvant chemotherapy as a standard of care for patients with TNBC,^37^ further investigation of PARP inhibitors with immunotherapy is in progress for patients with TNBC (talazoparib plus avelumab, NCT03330405; olaparib plus durvalumab, NCT03544125 and NCT03740893; niraparib plus ­dostarlimab, NCT04837209).

## Conclusion

In summary, despite not reaching the predefined threshold for the primary endpoint, in this phase II study that investigated neoadjuvant talazoparib monotherapy in patients with early-stage TNBC and germline *BRCA* mutations, the pCR rate was 45.8% in the evaluable population and 49.2% in the ITT population, and was comparable to observed pCR rates with anthracycline- and taxane-based chemotherapy combination regimens. The TRAEs in this study were consistent with the established safety profile of talazoparib.^20,21^

## Supplementary Material

oyad139_suppl_Supplementary_Material

## Data Availability

Upon request, and subject to review, Pfizer will provide the data that support the findings of this study. Subject to certain criteria, conditions and exceptions, Pfizer may also provide access to the related individual de-identified participant data. See https://www.pfizer.com/science/clinical-trials/trial-data-and-results for more information.
