# Supplementary material for: Neoadjuvant Talazoparib in Patients With Germline BRCA1/2 Mutation-Positive, Early-Stage Triple-Negative Breast Cancer: Results of a Phase II Study
Source: Oncologist. 2023 Jun 15;28(10):845–55. doi: 10.1093/oncolo/oyad139 (PMC10546823; doi:10.1093/oncolo/oyad139)
Supplement: oyad139_suppl_Supplementary_Material [file oyad139_suppl_supplementary_material.docx]

**Supplementary Material**

**Supplementary Table S1.** Patient disposition (ITT analysis population).

| **Patient disposition** | **Talazoparib (*N* = 61)** |
| --- | --- |
| **Number of patients, *n* (%)** |  |
| **Disposition phase: treatment^a^** |  |
| Entered | 61 (100.0) |
| Discontinued | 16 (26.2) |
| Completed | 45 (73.8) |
| **Disposition phase: safety follow-up^b^** |  |
| Entered | 49 (80.3) |
| Discontinued | 0 (0.0) |
| Completed | 49 (80.3) |
| **Disposition phase: long-term follow-up** |  |
| Entered | 58 (95.1) |
| Discontinued | 58 (95.1) |
| Study closed by sponsor | 55 (90.2) |
| Death | 2 (3.3) |
| Withdrawal by patient | 1 (1.6) |

Abbreviation: ITT, intent-to-treat.

The denominator to calculate percentages is *N*, the total number of patients in the ITT analysis set.

^a^One patient completed four treatment cycles prior to protocol-required surgery in order to obtain surgery prior to COVID-19 surgical restrictions.

^b^Includes patients who completed surgical follow-up.

**Supplementary Table S2.** Summary of breast cancer stage at initial diagnosis by pCR and RCB by ICR (ITT population).

| **pCR by ICR** | | | | | | | | | | |
| --- | --- | --- | --- | --- | --- | --- | --- | --- | --- | --- |
| **Number of patients, n** | | | | | | | | | |  |
| **Stage** | **pCR** | | **pPR** | | **NR** | **Missing^a^** | | **pCR rate (%)** | | |
| I | 9 | | 6 | | 1 | 4 | | 45.0 | | |
| II | 11 | | 8 | | 1 | 7 | | 40.7 | | |
| III/Other | 10 | | 2 | | 0 | 2 | | 71.4 | | |
| Total | 30 | | 16 | | 2 | 13 | | 49.2 | | |
| **RCB by ICR** | | | | | | | | | | |
| **Number of patients, n** | | | | | | | | | |  |
| **Stage** | | **0/I** | | **II** | | | **Missing^a^** | |  | |
| I | | 9 | | 7 | | | 4 | |  | |
| II | | 12 | | 8 | | | 7 | |  |  |
| III/Other | | 10 | | 2 | | | 2 | |  |  |
| Total | | 31 | | 17 | | | 13 | |  |  |

Abbreviations: ICR, independent central review; ITT, intent-to-treat; NR, no response; pCR, pathologic complete response; PD, progressive disease; pPR, pathologic partial response; RCB, residual cancer burden.

^a^Ten patients had PD and were counted in the Missing category. The counts of PD per stage at initial diagnosis were stage I=2 patients, stage II=6 patients, and stage III=2 patients (which by definition would be RCB III). Two patients did not have surgery for other reasons (early discontinuation and consent withdrawal), and one patient was unable to be assessed due to missing required axillary specimen.

**Supplementary Table S3.** Exposure to talazoparib (ITT population).

|  | **Talazoparib (*N* = 61)** |
| --- | --- |
| **Duration of treatment (weeks^a^)** |  |
| Mean | 23.3 |
| **Category (weeks^a^)** |  |
| <8 | 0 |
| 8-<16 | 3 (4.9) |
| 16-<20 | 3 (4.9) |
| 20-<24 | 10 (16.4) |
| ≥24^b^ | 45 (73.8) |
| **Overall relative dose intensity^c^** |  |
| Mean | 84.5 |

Abbreviation: ITT, intent-to-treat.

^a^Duration of exposure (weeks) was calculated as – (last dose date – first dose date + 1)/7.

^b^Per the protocol, patients were allowed to make up missed doses due to toxicities, which extended their duration of exposure.

^c^Overall relative dose intensity (%): this was calculated as – 100 x (overall cumulative dose)/(168 x starting dose of patient).
